# Suspension syndrome: a scoping review and recommendations from the International Commission for Mountain Emergency Medicine (ICAR MEDCOM)

**DOI:** 10.1186/s13049-023-01164-z

**Published:** 2023-12-09

**Authors:** Simon Rauch, Raimund Lechner, Giacomo Strapazzon, Roger B. Mortimer, John Ellerton, Sven Christjar Skaiaa, Tobias Huber, Hermann Brugger, Mathieu Pasquier, Peter Paal

**Affiliations:** 1 Institute of Mountain Emergency Medicine, Eurac Research, Bolzano, Italy; 2 Department of Anaesthesia and Intensive Care Medicine, Hospital of Merano (SABES-ASDAA), Merano-Meran, Italy; 3 Medical University Innsbruck, Innsbruck, Austria; 4 Department of Anesthesiology, Intensive Care Medicine, Emergency Medicine and Pain Therapy, Bundeswehr Hospital Ulm, Ulm, Germany; 5 International Commission for Mountain Emergency Medicine (ICAR MedCom), Zurich, Switzerland; 6 Corpo Nazionale del Soccorso Alpino E Speleologico (CNSAS), Milan, Italy; 7 Fresno Medical Education Program, Department of Family and Community Medicine, University of California, San Francisco, San Francisco, CA USA; 8 Division of Prehospital Services, Air Ambulance Department, Oslo University Hospital, Oslo, Norway; 9 Department of Anaesthesiology and Intensive Care Medicine, Salzkammergut Klinikum Vöcklabruck, Vöcklabruck, Austria; 10 Emergency Department, Lausanne University Hospital, Lausanne, Switzerland; 11 Department of Anaesthesiology and Intensive Care Medicine, St. John of God Hospital, Paracelsus Private Medical University, Salzburg, Austria

**Keywords:** Circumrescue collapse, Neurocardiac syncope, Harness, Suspension syndrome, Resuscitation, Rescue death

## Abstract

**Background:**

Suspension syndrome describes a multifactorial cardio-circulatory collapse during passive hanging on a rope or in a harness system in a vertical or near-vertical position. The pathophysiology is still debated controversially.

**Aims:**

The International Commission for Mountain Emergency Medicine (ICAR MedCom) performed a scoping review to identify all articles with original epidemiological and medical data to understand the pathophysiology of suspension syndrome and develop updated recommendations for the definition, prevention, and management of suspension syndrome.

**Methods:**

A literature search was performed in PubMed, Embase, Web of Science and the Cochrane library. The bibliographies of the eligible articles for this review were additionally screened.

**Results:**

The online literature search yielded 210 articles, scanning of the references yielded another 30 articles. Finally, 23 articles were included into this work.

**Conclusions:**

Suspension Syndrome is a rare entity. A neurocardiogenic reflex may lead to bradycardia, arterial hypotension, loss of consciousness and cardiac arrest. Concomitant causes, such as pain from being suspended, traumatic injuries and accidental hypothermia may contribute to the development of the Suspension Syndrome. Preventive factors include using a well-fitting sit harness, which does not cause discomfort while being suspended, and activating the muscle pump of the legs. Expediting help to extricate the suspended person is key. In a peri-arrest situation, the person should be positioned supine and standard advanced life support should be initiated immediately. Reversible causes of cardiac arrest caused or aggravated by suspension syndrome, e.g., hyperkalaemia, pulmonary embolism, hypoxia, and hypothermia, should be considered. In the hospital, blood and further exams should assess organ injuries caused by suspension syndrome.

## Background

Suspension syndrome describes a multifactorial cardio-circulatory collapse during passive hanging on a rope or in a harness system in a vertical or near-vertical position [1–3]. Although numerous cases have been reported, the incidence of suspension syndrome is not known [1, 4–7]. Since the first presentation of a case series in 1972 [2], its pathophysiology has been debated controversially [3, 8]. A widespread hypothesis assumes that blood pooling in the lower limbs leads to a reduction in cardiac preload, a consecutive decrease in cardiac output, tissue hypoperfusion and lastly loss of consciousness and cardiac arrest [2, 8, 9]. However, no study has ever proven this hypothesis and recent studies suggest a neurocardiogenic mechanism [10, 11]. The best measures for immediate aid by first responders is still debated and some recommendations advise against placing a casualty in a supine position after being rescued from suspension, hypothesising an acute right ventricular volume overload due to blood returning from the legs [2, 4, 12–14]. However, this hypothesis has also never been proven and is based on ‘expert opinion’ only [15]. The aim of the International Commission for Mountain Emergency Medicine (ICAR MedCom) was to perform a scoping review to identify all articles with original epidemiological and medical data to understand the pathophysiology and develop updated recommendations for the definition, prevention, and management of the suspension syndrome.

## Methods

This work comprises two distinct components. The first is a scoping review, and the second entails recommendations that were formulated through discussions within ICAR MedCom. We conducted a literature search in PubMed, Embase, Web of Science, and the Cochrane Library. We incorporated all articles available in the databases up to September 17, 2023 found with the keywords "suspension syndrome", "suspension trauma", "harness hang syncope", "harness hang syndrome", "rescue death", "harness suspension" and "harness syndrome" using the conjunction OR (RL) in order to identify all articles with epidemiological or original pathophysiologic data (e.g. vital signs, laboratory parameters, objective signs and symptoms). We also searched the bibliographies of articles relevant to this review and articles from the authors' personal databases (all). We excluded duplicates, articles on different scientific topics (= not eligible), articles that could not be retrieved in full text (= not available), trial registrations, conference abstracts, case reports without original data, reviews (no original data), articles in languages other than the authors' own (i.e., English, German, Italian), trial registrations, conference abstracts, letters, editorials, and short communications (SR, RL). Data were extracted independently by two authors (SR, RL); any disagreements were solved in discussion among the authors. Data was critically appraised using the National Heart, Lung and Blood Institute Study Quality Assessment Tools (SR, RL) [16]. The results pertinent to this work are synthesized in Table 1. The contents of the manuscript were developed by the author group and discussed within ICAR MedCom. Secondly, based on prior recommendations [17–22], the author group developed recommendations and presented them to all four commissions (i.e., air rescue, avalanche rescue, medical and terrestrial rescue) of the ICAR and discussed them at the 2019 ICAR meeting. The revised form was circulated in the ICAR MedCom list server for final review. Finally, the manuscript was approved for submission to a peer reviewed journal.

**Table 1:** Articles included for analysis

Author	Year	Study design	Sample Size	Key findings	Quality assessment
Patscheider [6]	1972	Observational autopsy study	5 (climbers died in the Tyrolean alps between 1957 – 1968)	Hypoxic changes of heart, liver and in one case kidney Protracted orthostatic shock is the cause of organ injury	Fair
Flora et al. [15]	1972	Summary of results from physiological experimental human studies with chest harness suspension presented at the 2^nd^ international Conference of Mountain Rescue physicians (original articles are not available)		Massive circulatory obstruction Increase of heart rate and reduction of pulse pressure Reduction of blood pressure Drop of central venous pressure Reduction of renal function ECG changes Presyncopal syndroms in subjects	Poor
Flora et al. [2]	1972	Case series with pathophysiologic parameters	23 (10 died, 13 survived)	Reported pathophysiological conditions: acute kidney injury, hypoxic heart muscle damage, muscle necrosis and dyspnoea Arm plexus damage in chest harness after 10 to 20 min of free suspension, if no foot loops were used Death occurred regularly after suspension longer than two hours	Fair
Stuehlinger et al. [24]	1972	Observational physiologic human study	10, two trial runs	Hanging in a chest harness was aborted after 8 to 22 min due to pallor, cold sweat, nausea and paraesthesia in the upper extremities Hanging in a sit harness was aborted in 3 cases after 22 to 28 min due to clinical symptoms Tachycardia and hypertension due to sympathetic activation Indirect signs of peripheral blood pooling Sinus arrythmia and extrasystoles after placement in horizontal position after trial, which led to the assumption of sudden cardiac arrest due to horizontal placement after hanging Glomerular filtration and renal plasma flow drops already after only 8 min, with further drop while hanging	Fair
Orzech et al. [25]	1987	Observational physiologic human study and review	13 (each with body belt, chest harness, full body harness)	Suspension tolerance in a body belt or chest harness alone is significantly lower than in a full body harness, due to pain and difficulty of breathing during hanging	Fair
Roeggla et al. [26]	1996	Observational physiologic human study	6 (each with chest harness alone or sit harness)	Decrease of vital capacity, forced expiratory volume and cardiac output in chest harness suspension	Good
Madsen et al. [27]	1998	Observational physiologic human study	9 knee strap suspension 79 50° head up tilt table	11% developed symptoms with knee strap, 87% developed symptoms with 50° head up tilt Longer tolerance of suspension with support strap under knees due to preserved venous return Initial heart rate and blood pressure increase followed by a drop with onset of presyncopal symptoms Reduction of central blood volume during head up tilt	Fair
Shamsuzzaman et al. [28]	1998	Observational physiologic human study	13 (each on tilt table and tilted suspension)	One vasovagal reaction during “suspension” Engagement of antigravity muscles have effects on sympathetic vasoconstriction and cardiovascular responses During suspension reduction of cardiac output and stroke volume, tachycardia and hypertension	Good
Rollnik et al. [29]	2001	Observational physiologic human study	14	Suspension in supine position (one sling around the thorax and one sling around the thighs) caused only moderate heart rate changes, which were significantly lower compared to vertical suspension Vertical suspension with a sling placed under the armpits was tolerated only 2—4 min	Fair
Seddon et al. [4]	2002	National survey	Not applicable	No suspension syndrome within 5.8 million on-rope hours by rope access technicians qualified by IRATA (International Rope Access Trade Association)	Fair
Pisati et al. [30]	2007	Case report with discussion of pathophysiologic parameters	2	Prolonged hanging in a sit harness caused pulmonary thrombo-embolism and thrombosis of femoral artery, most likely due to compression by harness groin straps	Poor
Turner et al. [31]	2008	Observational physiologic human study	40 (each on front and back attachment) 28 (harness with leg support for a more horizontal suspension position)	Harness with leg support doubled suspension time Body weight reduced suspension time with back attachment	Good
Ruhrmann et al. [32]	2010	National survey among height rescue organizations	68 questionnaires	3 cases of suspension syndrome in 131 datasets	Poor
Wharton et al. [7]	2011	Case report with pathophysiologic parameters	1	Suspension > 4 h Rhabdomyolysis and acute kidney injruy	Poor
Hsiao et al. [33]	2012	Observational physiologic human study	37	Suspension tolerance between 5 and 56 min Static harness fit does not correlate with suspension tolerance Improperly conformed / sized harness could increase risk of suspension syndrome Rear attachment with angle > 35° between torso and suspension line has shorter suspension time	Good
Hsiao et al. [34]	2013	Observational physiologic human study and Fourier analysis of torso scans	216	different harness sizes are necessary for optimal fit Harness fit is partly dependent on gender Harness adjustment is necessary for clothing and other equipment	Good
Goossens et al. [35]	2014	Case report	1	Combination of heat stroke and suspension syndrome after 1 h of suspension Unconsciousness, rhabdomyolysis, and acute kidney injury	Poor
Lanfranconi et al. [10]	2017	Observational physiologic human study	40	Syncope in 10% with drop of systolic blood pressure, tachycardia just before syncope and bradycardia during recovery Onset of syncope from aprupt to minutes after first clinical signs Decrease of cerebral oxygenated hemoglobin before syncope	Good
Beverly et al. [36]	2019	Observational physiologic human study	18, with two trial runs	Tolerance of rear attachment hanging is significantly lower than front attachment (shorter duration of hanging, more trial terminations) 25% suffered symptoms which lead to trial termination, one near syncope Bradycardia in three subjects who terminated trial early Weak laboratory signs of muscle damage with dorsal attachment (significant aspartate aminotransferase increase)	Fair
Lanfranconi et al. [37]	2019	Observational physiologic human study	40	Syncope in 10% with decrease of arterial oxygen saturation and a drop of systolic blood pressure Short suspension tolerance participants (no syncope) had a changed of breathing pattern (increase of tidal volume, decrease of frequency, increase in respiratory elastic power) stress induced hypertension (sympathetic activation)	Good
Rauch et al. [11]	2019	Observational physiologic human study	20	Sudden pre-syncope in 30% Vagal mechanism leads to loss of consciousness (bradycardia and drop in systolic blood pressure) Time to pre-syncope is unpredictable	Good
Rauch et al. [38]	2019	Observational physiologic human study	20	Free hanging leads to rapid venous pooling in the limbs Most important preventive measure might be constant leg movement Pulmonary embolism might be preventable cause of death	Good
Baszczynski [39]	2022	Observational manikin study	4 industrial climbing harnesses	Industrial climbing harnesses, especially with rear attachment, have a high risk of excessive local pressure	Poor

Based on our findings we developed recommendations on prevention, diagnosis and treatment of the suspension syndrome, we graded them according to the Grading System of the American College of Chest Physicians [23].

## Results

### Literature search

**Fig. 1:**
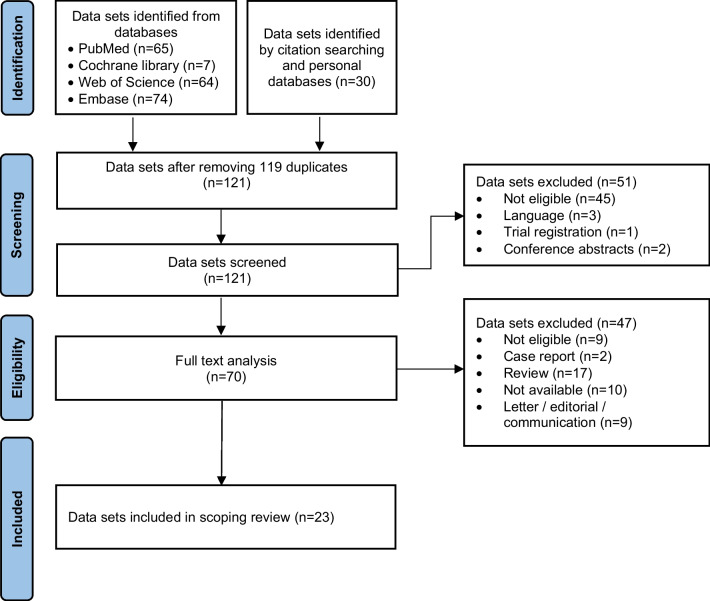
PRISMA flow diagram depicting the study selection process

Of the 121 studies returned from search, 70 met inclusion criteria after screening the titles and abstracts. On full-text review, 47 studies were excluded (Fig. 1) and finally, 23 articles were included (Table 1).

### Description of *studies*

The 23 studies included were divided into two themes based on their characteristics: Epidemiology and Pathophysiology. Two studies reported epidemiological data from surveys while 21 studies were on pathophysiology. Studies on pathophysiology ranged from autopsy studies [1] to manikin [1] and small interventional/observational [14] human studies to case series or reports including pathophysiological data [5]. Some studies on pathophysiology also assessed the effect of different harness types.

#### Epidemiology

Exact data on the epidemiology of suspension syndrome is lacking. Since the use of sit harnesses, the incidence of death from suspension syndrome is very rare. In an industrial setting, there have been no fatalities or syncopal events published; this was confirmed by a negative enquiry to a large trade body in the UK by Seddon in 2002, which found no suspension syndrome within 5.8 million on-rope hours by rope access technicians qualified by IRATA (International Rope Access Trade Association) [4]. A small survey among height rescue organizations identified 3 cases of suspension syndrome, but without stating observational periods and describing symptoms or severity [32]. It is postulated that awareness, health and safety regulations and training in extrication techniques have mitigated against serious adverse events.

#### Pathophysiology

Until recently, a widespread hypothesis assumed that blood pooling in the lower limbs prompts a reduction in cardiac preload and subsequently a decrease in cardiac output and tissue perfusion, eventually leading to loss of consciousness, liver and kidney injury [15] and cardiac arrest [2]. However, an unequivocal causal relationship between blood pooling and cardiac arrest has never been proven and more recent studies support the hypothesis that a neurocardiogenic mechanism is responsible for the sudden reduction in cerebral perfusion and loss of consciousness in suspension syndrome. Experimental studies in healthy participants hanging in a harness system showed venous pooling starting from bigger veins and progressively involving small venous vessels in the lower extremities [11, 38]. However, despite this sequestration of blood in the lower limbs due to the force of gravity and the absence of muscle activity, no relevant effects on systemic hemodynamic parameters were found (i.e., heart rate, blood pressure, stroke volume), besides occasionally a mild tachycardia and hypertension which was attributed to discomfort and sympathetic activation [10, 11, 15, 24, 27, 28, 37]. This persisted until a sudden drop in heart rate, blood pressure and stroke volume, similar to a neurocardiogenic syncope with a vasodilatory and cardio-inhibitory response, which led to a decreased cerebral oxygenation [10, 11, 26–28, 36–38]. Simultaneously, symptoms of pre-syncope such as dizziness, light-headedness, pale skin, sweating, blurred vision and nausea occurred [11, 25, 27, 38]. The absence of distinct compensatory tachycardia and reduced stroke volume, which are usually seen when cardiac preload is significantly reduced, suggest that the above mentioned traditional pathophysiological hypothesis of suspension syndrome is incorrect. However, the exact mechanism leading to the neurocardiogenic reflex and the role of orthostatic stress (i.e., blood pooling) in this context are unknown [40]. No change in baroreceptor-sensitivity, a possible mechanism leading to a neurocardiogenic syncope, was found in the minutes before the pre-syncope [11]. The Bezold-Jarisch reflex, a cardioinhibitory reflex causing bradycardia, vasodilation, and hypotension, could be implicated in suspension syndrome. However, the Bezold-Jarisch reflex originates from inhibitory mechanoreceptors in the left ventricle stimulated by a poor filling, which was not found in experimental studies [11]. Altered sensation in the lower limbs, pain and the inability to move the legs, likely caused by compression of the sciatic nerve by the harness straps [11, 26], could significantly contribute to the development of the neurocardiogenic syncope [41].

**Fig. 2:**
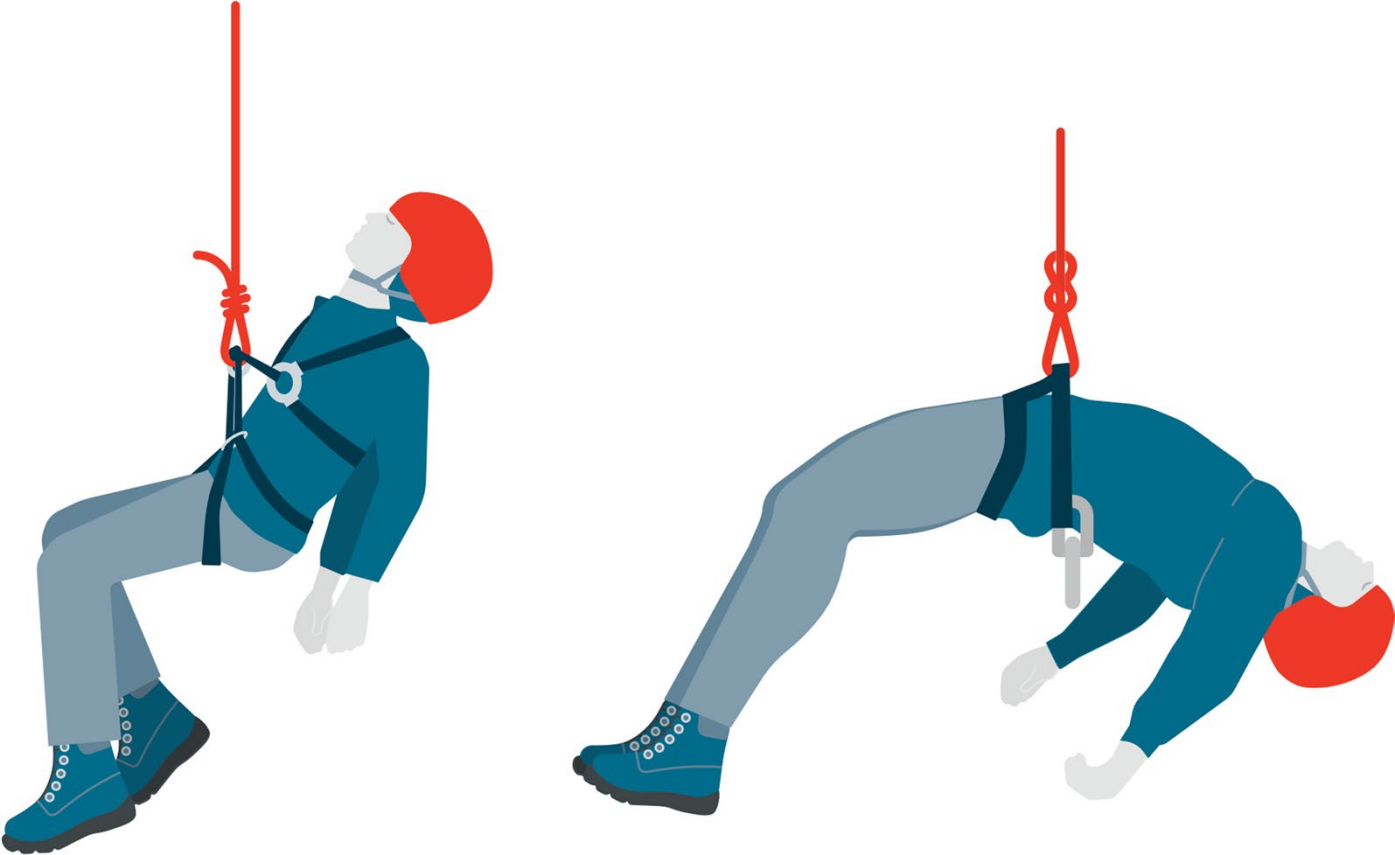
Hanging motionless on a rope precludes a horizontal position after loss of consciousness whether the attachment point is at the level of the chest (left) or hip (right)

During syncope, cerebral perfusion is quickly restored after an induced fall to the horizontal position. In contrast, hanging motionless on a rope precludes a horizontal position after loss of consciousness whether the attachment point is at the level of the hip as for a sit harness or at a higher level as with a chest harness (Fig. 2). The resulting positions can lead to a further decrease of organ perfusion that can be followed by death. Moreover, in the scenario of an unconscious patient suspended on a rope, airway obstruction caused by hyperflexion or hyperextension of the neck can further contribute to a potentially fatal outcome [42].

Studies showed an inter-individual variability in time to occurrence of the first symptoms of pre-syncope. This can be as short as a few minutes but can be up to several hours [2, 10, 25, 33]. In addition to the unpredictable pathophysiological development, the symptoms of pre-syncope can also occur suddenly without a prodromal stage [11]. The level of pain might be an important factor contributing to the (time to the) vagal event [11, 26]. Gender does not seem to have any effect, whereas increased body weight seems to decrease time suspension is tolerated [31, 33]. Exercise before suspension could increase the susceptibility and decrease the time until suspension syndrome occurs [11].

Prolonged suspension can have additional effects. Tissue hypoperfusion and hypoxia may lead to cell lysis, particularly in muscle tissue [7, 35, 36]. Rhabdomyolysis may cause hyperkalaemia, which may lead to a ventricular arrhythmia as well as acute kidney injury. Accidental hypothermia can be associated with prolonged hanging and can further affect the course of suspension syndrome. Thromboembolic events have been associated with suspension syndrome due to venous stasis but was not associated with any of the deaths [30]. Paraesthesia and other neurologic lesions have been reported [2, 25].

##### Harness types

The type of harness has an influence on hanging. When hanging in a belt (without leg loops) or in a chest harness alone, very severe pain, pressure paralysis of the brachial nerves and a marked decrease in cardiopulmonary parameters due to restricted chest and diaphragm motion can occur rapidly and was associated with a higher incidence of suspension syndrome compared to today. [24–26, 29]. These types of attachment are therefore considered obsolete and must be distinguished from the suspension syndrome in modern sit harnesses, which became increasingly widespread from the early 1970s onwards.

Suspension in harnesses with a dorsal attachment, as is often used in industrial climbing, is also associated with lower suspension tolerance in some but not all studies [36]. The hanging position is more vertical and a femoral vein compression, which may reduce venous return, is possible [39]. In addition, self-rescue is more difficult. However, the dorsal attachment is fundamentally different from the harness systems used in speleology and mountain sports. Anthropometric data, suspension angle and suspension tolerance from such harness systems can therefore not be easily transferred to sit harness systems with ventral attachment points [33]. An improperly fitted and sized belt can increase the risk of developing a suspension syndrome, for example by causing a pain-triggered vagal syncope due to nerve compression [34].

## Discussion

To the best of our knowledge, this is the first scoping review on suspension syndrome. The literature search identified surveys on epidemiology of suspension syndrome and studies delving into its pathophysiology. Although precise and up-to-date epidemiological data remain elusive, we uncovered several studies of fair quality shedding light on the condition's pathophysiological aspects. In essence, suspension is shown to cause venous pooling, yet this does not appear to trigger significant hemodynamic repercussions. The loss of consciousness can be attributed to a neurocardiogenic mechanism. Notably, comprehensive and credible data are exclusively available from experimental human studies, as randomized controlled interventional studies are absent. These experimental studies predominantly center on the underlying pathogenetic mechanisms during the early stages of suspension syndrome. Ethical considerations constrain the evaluation of prolonged exposure beyond the onset of pre-syncopal symptoms and signs, leaving the outcomes of later stages, including cardiac arrest, shrouded in uncertainty.

Our scoping review did not yield experimental studies on the prevention, diagnosis, and treatment of suspension syndrome. Nevertheless, given the clinical significance of these aspects, we next provide discussions informed by the pathophysiological data gleaned from the scoping review and from treatment recommendations for other conditions, e.g., cardiac arrest.

Furthermore, it is worth noting that the terminology, definition, and classification of suspension syndrome in the literature display significant heterogeneity. As a result, we propose a framework to address these aspects.

### Definitions and classification

All terms with harness, such as “harness hang syncope” or “harness syndrome” should be avoided, as being in a harness is not a prerequisite to develop suspension syndrome. Also, all terms with trauma as “suspension trauma” are inappropriate, because often there is no or only minimal trauma. The term suspension syndrome includes (near) syncope, death on the rope, death after rescue and cases of subacute renal failure from rhabdomyolysis after rescue (Table 1). We propose the following classification of suspension syndrome, which is based on the severity of the symptoms, different pathophysiology, and the time course of the suspension syndrome:

**Table:** 

1. Acute suspension syndrome
a) Near suspension syncope (characterized by light-headedness, dizziness, confusion,
pale skin, cold sweating, warmth / hot flashes, blurred vision or nausea, bradycardia)
b) Suspension syncope
c) Suspension cardiac arrest
d) Post-suspension cardiac arrest within 60 min after rescue
2. Subacute suspension syndrome
a) Sensory or motoric deficit in the legs persisting for > 24 h after rescue
b) End organ dysfunction, in particular rhabdomyolysis-associated acute kidney injury
c) Cardiac arrest > 60 min after rescue

None of the signs and symptoms must be attributable to trauma or any other medical condition (e.g., trauma, hypothermia, hypoglycemia).

### Diagnosis and treatment

As suspension syndrome usually occurs in terrain where there is a risk of falling, it is important to ensure rescuer safety. The overall treatment principles follow normal management of vaso-vagal symptoms and, if critical, standard advanced life support (ALS) protocols [43, 44]. Treatment of pre-syncope and syncope should be initiated immediately on extrication of the patient from the rope. The horizontal supine position to improve cerebral blood flow is recommended. Previous recommendations suggested not laying a patient down abruptly, but there is no evidence to support this [1, 4, 9, 45, 46]. If victims cannot be immediately extricated from the rope, preventive measures, as described above, should be performed.

For an early diagnosis and a prompt intervention it is important to know and pay attention to the typical pre-syncopal signs and symptoms: light-headedness, dizziness, confusion, pale skin, cold sweating, warmth/hot flashes, blurred vision or nausea, bradycardia [11, 31, 36, 47]. The orthostatic tolerance varies individually, and symptoms of pre-syncope may occur at any time during passive hanging on a rope.

First diagnostic measures on site after recovery from hanging include monitoring of pulse and respiration, blood pressure, peripheral oxygen saturation (SpO_2_) and electrocardiogram (ECG). The ECG should be analysed for signs of hyperkalaemia (peaked T waves, flat/absent P waves, broad QRS, sine waves) and arrhythmias (bradycardia, ventricular fibrillation) [44]. Portable ultrasound could be useful for detection of deep venous thrombosis or right ventricular dilation from pulmonary embolism and extended focused assessment with sonography for trauma (eFAST) [48–50]. Continuous full monitoring including body core temperature after recovery from hanging and during transport is recommended. Upon hospital admission, blood tests including liver and kidney function tests, creatine kinase (CK) and myoglobin, along with blood gas analysis are recommended to detect for possible electrolyte, acid base disturbances and rhabdomyolysis [7, 42, 51]. Monitoring of urinary output is also recommended. Consider a CT-scan to detect or exclude accompanying injuries.

Cardiac arrest is the end stage of suspension syndrome, and it may occur when an incapacitated patient suspended in a near-vertical position drops in blood pressure and heart rate below critical levels of organ perfusion. Emergency treatment is immediate extrication to flat ground and the initiation of standard ALS protocols. Rhabdomyolysis from lower extremity ischaemia should be suspected in cases where there has been more than 2 h of passive suspension [2, 3, 7]. Hyperkalaemia and kidney failure could develop in both the conscious and unconscious patient. There are few cases of death occurring directly after rescue; it is speculated that this may be an effect of potassium returning from the legs [3]. Diagnosis and treatment of suspected rhabdomyolysis should follow standard protocols. In the case of cardiac arrest following suspension, hyperkalemia and pulmonary embolism should be considered and treated empirically, particularly after prolonged suspension [3, 7]. Drugs that increase serum potassium should be avoided, e.g., succinylcholine. Other likely reversible causes of death in cardiac arrest are hypoxia from airway obstruction during hanging and hypothermia [38]. Those reversible causes of cardiac arrest should be treated according to current guidelines [44]. Symptoms of nerve compression from the harness do occur [11]. No specific therapy is available and recovery from nerve compression requires time.

### Prevention

Expert opinion suggests that anyone hanging passively in a harness or indeed held in an upright position without a harness is at risk of developing a suspension syndrome [3].

The most important preventive measure for the climber is having proper equipment and knowledge on how to use the former, especially personal rope skills [3]. Climbers and rescuers should be aware that loss of consciousness and cardiac arrest can occur at any time when a person is hanging motionless on rope. Once a person is stuck on rope, they must be brought down immediately. Therefore, rope work should never be conducted alone. The climber’s or worker’s team members must have a plan for recovering a person quickly if they cannot escape the situation themselves.

If the person cannot be recovered, some steps may assist the person. Suspension syndrome is a consequence of passive, immobile suspension, not just being suspended on rope. Active movement of the legs maintains and restores venous return to the heart [38]. A rock, crevice or house wall or a clipped step sling can also be used as an abutment to activate the muscle pump. A backpack should be taken off the back and hung, for example, at the attachment point to make hanging more comfortable. To alleviate muscle work needed to sit comfortably over time and increase comfort, the upper body can be stabilised by a sling which is passed under the armpits and clipped into the rope. Care must be taken to ensure that the sling does not cut into the body painfully. Suspended persons should be encouraged to continue moving the legs. Alternatively, a strap or sling under the knees may be used to raise up the legs closer to heart level [27, 29, 31]. Alternatively, the legs may be lifted by a rescuer. This may be especially important if the suspended person feels at risk of losing consciousness for any reason and would not be able to continue moving their legs. An added benefit of the supported legs is that it takes some pressure of the harness itself which may reduce effects of nerve compression [4].

A person stuck on a rope is an emergency. It is appropriate to activate an emergency response early. Rescue teams should be properly equipped and trained to recover a person stuck on a rope in the shortest possible time.

A hanging test with the equipment should be performed to best adjust the harness and find the least painful hanging possible while suspended before first use of the harness. Well-fitting leg loops on a modern harness do not restrict blood flow of the femoral vessels [3]. The decision whether to remove a harness during the rescue and treatment of the patient should therefore be based purely on the technical aspects of rescue and not on medical aspects.

## Research implications

The incidence and the relevance of suspension syndrome should be better described, e.g., through an international suspension syndrome registry. Also, the pathophysiology should be better assessed, e.g., whether a grading of suspension syndrome based on pathophysiological parameters can be achieved.

## Limitations

Suspension Syndrome has a very low incidence; severe cases are especially rare. There are only a few case reports, some of which could not be thoroughly analysed because of incomplete data. Certain literature from the era of the initial description of suspension syndrome is no longer accessible. Consequently, the pathophysiological insights presented therein cannot be integrated into the current discussion. No inter-rater reliability was calculated for the assessment of the results of the literature search. Comprehensive data are available from experimental human studies and focus on the underlying pathogenetic mechanisms during the early stages of suspension syndrome. Randomized controlled interventional studies on suspension syndrome are absent. Regarding the quality assessment based on NHLBI grading, the studies often could not be clearly assigned to the categories specified by the NHLBI and usually had only small sample sizes.

## Conclusions

Suspension syndrome is a rare entity. It is caused by hanging suspended in a harness. A neurocardiogenic reflex may lead to bradycardia, arterial hypotension, and cardiac arrest. Concomitant causes, such as pain from being suspended, traumatic injuries and accidental hypothermia may contribute to the development of suspension syndrome. Preventive factors include using a well-fitting sit harness, which does not cause discomfort while being suspended, and activating the muscle pump of the legs. Expediting help to extricate the suspended person is key. In a peri-arrest situation, the person should be positioned supine and standard ALS should be initiated immediately. Reversible causes of cardiac arrest caused by suspension syndrome, e.g., hyperkalaemia and pulmonary embolism, should be considered. In the hospital, blood and further exams should assess organ injuries caused by suspension syndrome. An international registry on suspension system is warranted to assess its incidence, pathophysiology, and outcome.

## Recommendations

Based on our scoping review, discussion with the ICAR MedCom and to give guidance on the most important questions regarding the suspension syndrome, the following recommendations on prevention, diagnosis and treatment of the suspension syndrome have been developed and graded according to the Grading System of the American College of Chest Physicians [23].

**Table:** 

No.	Recommendation	Grade
1	Rope work should be done only with proper equipment and knowledge on how to use it correctly. Rope work should never be conducted alone	1C
2	Persons suspended in a harness should be rescued as soon as possible, even if the casualty is asymptomatic, as time to near or actual syncope and potentially cardiac arrest is variable and unpredictable [11]	1B
3	While awaiting rescue, persons suspended freely on a rope should move their legs to reduce venous pooling [11, 38]	2B
4	If no adjoining structures are in reach, foot loops should be used to step in and increase the activation of the muscle pump [1, 3, 11, 42]	2B
5	If the casualty is no longer able to act and it is safe to do so, the first rescuer reaching the casualty should raise the victim’s legs to create a more horizontal position while measures are taken to lower the patient to the ground [11, 42]	2C
6	Once the casualty is on the ground, the casualty should be positioned supine. Assessment and treatment should follow standard advanced life support algorithms. Reversible causes of cardiac arrest, including hyperkalaemia and pulmonary embolism, should be considered, and managed appropriately [1, 3, 11, 42, 43, 45, 46]	1A
7	After prolonged hanging (> 2 h), patients are at risk of developing hyperkalaemia and acute kidney injury and should therefore be transported to a hospital with the capability of performing emergent renal replacement therapy [2, 3, 7]	2C

## Data Availability

All data generated or analysed during this study are included in this published article.

## Abbreviations

ALS: Advanced life support
CT: Computer tomography
ECG: Electrocardiogram
eFAST: Extended focused assessment with sonography for trauma
ICAR: International Commission for Alpine Resuce
ICAR MedCom: International Commission for Alpine Rescue, Medical Commission or the Medical commission for mountain emergency medicine
SpO_2_: Peripheral oxygen saturation

## References

[1] Pasquier M, Yersin B, Vallotton L, Carron PN (2011). Clinical update: suspension trauma. Wilderness Environ Med.

[2] Flora G, Holzl H. Fatal and non-fatal accidents involving falls into the rope. Paper of the Second International Conference of Mountain Rescue Doctors, Innsbruck (Austria) 1972.

[3] Mortimer RB (2011). Risks and management of prolonged suspension in an Alpine harness. Wilderness Environ Med.

[4] Seddon P. Harness suspension: review and evaluation of existing information. Norwich: Health and Safety Executive 2002.

[5] Amphoux M, editor Hanging after a fall: an extremely urgent rescue1998; Wuppertal, Germany.

[6] Patscheider H. Pathologico-anatomical examination results in the case of death caused by hanging on the rope. Paper of the Second International Conference of Mountain Rescue Doctors, Innsbruck (Austria) 1972.

[7] Wharton DR, Mortimer RB (2011). Rhabdomyolysis after prolonged suspension in a cave. Wilderness Environ Med.

[8] Lee C, Porter KM (2007). Suspension trauma. Emerg Med J.

[9] Adisesh A, Lee C, Porter K (2011). Harness suspension and first aid management: development of an evidence-based guideline. Emerg Med J.

[10] Lanfranconi F, Pollastri L, Corna G, Bartesaghi M, Novarina M, Ferri A (2017). The elusive path of brain tissue oxygenation and cerebral perfusion in harness hang syncope in mountain climbers. High Alt Med Biol.

[11] Rauch S, Schenk K, Strapazzon G, Dal Cappello T, Gatterer H, Palma M, et al. Suspension syndrome: a potentially fatal vagally mediated circulatory collapse—an experimental randomized crossover trial. Eur J Appl Physiol. 2019. 10.1007/s00421-019-04126-5 PMC6517360 30895459

[12] Raynovich B, Rwaili FT, Bishop P. Dangerous suspension. Understanding suspension syndrome & prehospital treatment for those at risk. JEMS: J Emerg Med Serv. 2009;34(8):44–51, 3; quiz 3. 10.1016/S0197-2510(09)70215-3 19665660

[13] Drew R. Suspension trauma. Can J Emerg Nurs. 2020.

[14] Petrone P, Espinoza-Villalobos S, Baltazar GA, Søreide K, Stright A, Brathwaite CEM (2021). Fatal and non-fatal injuries due to suspension trauma syndrome: a systematic review of definition, pathophysiology, and management controversies. World J Emerg Med.

[15] Flora GM, R.; Dittrich, P.; Stuhlinger, W. Hanging tests—conclusions for the mountaineer. Paper of the Second International Conference of Mountain Rescue Doctors (Austria) German to English translation by HSE Language Services Transl No 16372(I)1972.

[16] National Heart L, and Blood Institute (NHLBI). Study Quality Assessment Tools. 2021. Available from: https://www.nhlbi.nih.gov/health-topics/study-quality-assessment-tools.

[17] Pasquier M, Strapazzon G, Kottmann A, Paal P, Zafren K, Oshiro K (2023). On-site treatment of avalanche victims: scoping review and 2023 recommendations of the international commission for mountain emergency medicine (ICAR MedCom). Resuscitation.

[18] Blancher M, Albasini F, Elsensohn F, Zafren K, Hölzl N, McLaughlin K (2018). Management of multi-casualty incidents in mountain rescue: evidence-based guidelines of the international commission for mountain emergency medicine (ICAR MEDCOM). High Alt Med Biol.

[19] Sumann G, Moens D, Brink B, Brodmann Maeder M, Greene M, Jacob M, et al. Multiple trauma management in mountain environments - a scoping review: evidence based guidelines of the International Commission for Mountain Emergency Medicine (ICAR MedCom). Intended for physicians and other advanced life support personnel. Scand J Trauma Resuscitation Emerg Med. 2020;28(1):117. 10.1186/s13049-020-00790-1 PMC7737289 33317595

[20] Roy S, Soteras I, Sheets A, Price R, Oshiro K, Rauch S (2021). Guidelines for mountain rescue during the COVID-19 pandemic: official guidelines of the international commission for alpine rescue. High Alt Med Biol.

[21] Lugnet V, McDonough M, Gordon L, Galindez M, Mena Reyes N, Sheets A, et al. Termination of cardiopulmonary resuscitation in mountain rescue: a scoping review and ICAR MedCom 2023 recommendations. High Alt Med Biol. 2023. 10.1089/ham.2023.0068 37733297

[22] Ellerton J, Milani M, Blancher M, Zen-Ruffinen G, Skaiaa SC, Brink B (2014). Managing moderate and severe pain in mountain rescue. High Alt Med Biol.

[23] Guyatt G, Gutterman D, Baumann MH, Addrizzo-Harris D, Hylek EM, Phillips B (2006). Grading strength of recommendations and quality of evidence in clinical guidelines: report from an American College of Chest Physicians Task Force. Chest.

[24] Stuhlinger WD, P; Flora, G; Margreiter, R. Circulatory and renal function changes in test subjects suspended from the upper half of the body. Paper of the Second International Conference of Mountain Rescue Doctors, Innsbruck (Austria) 1972.

[25] Orzech M GM, Brickley J, Salerno M, Seaworth J. test program to evaluate human response to prolonged motionless suspension in three types of fall protection harnesses. . In: Armstrong Aerospace Medical Research Laboratory W-PAFB, editor. 1987.

[26] Roeggla M, Brunner M, Michalek A, Gamper G, Marschall I, Hirschl MM (1996). Cardiorespiratory response to free suspension simulating the situation between fall and rescue in a rock climbing accident. Wilderness Environ Med.

[27] Madsen P, Svendsen LB, Jørgensen LG, Matzen S, Jansen E, Secher NH (1998). Tolerance to head-up tilt and suspension with elevated legs. Aviat Space Environ Med.

[28] Shamsuzzaman AS, Sugiyama Y, Kamiya A, Fu Q, Mano T. Head-up suspension in humans: effects on sympathetic vasomotor activity and cardiovascular responses. J Appl Physiol (Bethesda, Md : 1985). 1998;84(5):1513–9. 10.1152/jappl.1998.84.5.1513 9572793

[29] Rollnik JD, Witt K, Hänert W, Rix W, Schwindt M (2001). Rescue lifting system (RLS) might help to prevent death after rescue from immersion in cold water. Int J Sports Med.

[30] Pisati G, Cerri S, Achille G, Rossi G, Lorenzi G (2007). Vascular thrombosis and pulmonary thrombo-embolism due to harness suspension. Med Lav.

[31] Turner NL, Wassell JT, Whisler R, Zwiener J (2008). Suspension tolerance in a full-body safety harness, and a prototype harness accessory. J Occup Environ Hyg.

[32] Ruhrmann S, Lutz M, Uhle F, Rehmann H, Haverney F, Weigand M, et al. Medizinische Versorgung in der Höhenrettung. Notfall + Rettungsmedizin. 2010;13(6):458–64.

[33] Hsiao H, Turner N, Whisler R, Zwiener J (2012). Impact of harness fit on suspension tolerance. Hum Factors.

[34] Hsiao H (2013). Anthropometric procedures for protective equipment sizing and design. Hum Factors.

[35] Goossens L, Vander Laenen M, Boer W (2014). Abstracts presented at the fifth symposium of the Belgian Society of Emergency and Disaster Medicine (BESEDIM); Abstract Nr: 24 Hanging by a thread. Acta Clin Belg.

[36] Beverly JM, Zuhl MN, White JMB, Beverly ER, VanDusseldorp TA, McCormick JJ (2019). Harness suspension stress: physiological and safety assessment. J Occup Environ Med.

[37] Lanfranconi F, Ferri A, Pollastri L, Bartesaghi M, Novarina M, De Vito G (2019). Impact of hanging motionless in harness on respiratory and blood pressure reflex modulation in mountain climbers. High Alt Med Biol.

[38] Rauch S, Schenk K, Gatterer H, Erckert M, Oberhuber L, Bliemsrieder B (2020). Venous pooling in suspension syndrome assessed with ultrasound. Wilderness Environ Med.

[39] Baszczyński K (2022). Effect of safety harness design on the pressures exerted on the user's body in the state of its suspension. Int J Occup Saf Ergon.

[40] Mosqueda-Garcia R, Furlan R, Tank J, Fernandez-Violante R (2000). The elusive pathophysiology of neurally mediated syncope. Circulation.

[41] Chen-Scarabelli C, Scarabelli TM (2004). Neurocardiogenic syncope. BMJ (Clin Res ed).

[42] Kolb JJ, Smith EL. SUSPENSION SHOCK. Redefining the diagnosis and treatment of suspension trauma. JEMS: J Emerg Med Serv. 2015;40(6):48–51. 26263737

[43] Soar J, Böttiger BW, Carli P, Couper K, Deakin CD, Djärv T (2021). European resuscitation council guidelines 2021: adult advanced life support. Resuscitation.

[44] Lott C, Truhlář A, Alfonzo A, Barelli A, González-Salvado V, Hinkelbein J (2021). European resuscitation council guidelines 2021: cardiac arrest in special circumstances. Resuscitation.

[45] Thomassen O, Skaiaa SC, Brattebo G, Heltne JK, Dahlberg T, Sunde GA (2009). Does the horizontal position increase risk of rescue death following suspension trauma?. Emerg Med J.

[46] Adisesh A RL, Codling A, Harris-Roberts J, Lee C, Porter K. Evidence-based review of the current guidance on first aid measures for suspension trauma. . In: Executive HaS, editor. Norwich, UK; 2009.

[47] Weber SA, McGahan MM, Kaufmann C, Biswas S (2020). Suspension trauma: a clinical review. Cureus.

[48] Fitzgibbon JB, Lovallo E, Escajeda J, Radomski MA, Martin-Gill C (2019). Feasibility of out-of-hospital cardiac arrest ultrasound by ems physicians. Prehosp Emerg Care.

[49] Robinson AE, Simpson NS, Hick JL, Moore JC, Jones GA, Fischer MD, et al. Prehospital ultrasound diagnosis of massive pulmonary embolism by non-physicians: a case series. Prehosp Emerg Care. 2022:1–6. 10.1080/10903127.2022.2113190 35952352

[50] Scharonow M, Weilbach C (2018). Prehospital point-of-care emergency ultrasound: a cohort study. Scand J Trauma Resuscitation Emerg Med.

[51] Mortimer RB, Zafren K (2020). Evidence-based versus myth-based treatment of suspension syndrome. Wilderness Environ Med.

